# What's new in heart failure? November 2025

**DOI:** 10.1002/ejhf.70089

**Published:** 2026-01-04

**Authors:** Mert Tokcan, Julian Hoevelmann, Philipp Markwirth, Bernhard Haring

**Affiliations:** ^1^ Department of Internal Medicine III Saarland University Hospital Homburg/Saar Germany; ^2^ HOMICAREM (HOMburg Institute of CArdioREnalMetabolic Medicine), Medical Faculty Saarland University Saarbrücken Germany; ^3^ Cape Heart Institute, Faculty of Health Sciences University of Cape Town Cape Town South Africa; ^4^ Department of Epidemiology and Population Health Albert Einstein College of Medicine Bronx NY USA; ^5^ Department of Medicine IV Clinic Hietzing, Vienna Healthcare Group Vienna Austria

In this column, we want to provide clinicians and researchers with short and concise summaries of recently published studies in the *European Journal of Heart Failure* that we think may be of particular relevance to heart failure (HF) specialists (*Figure* *1*). Key topics of this issue include characteristics and outcomes of patients with HF and history of malignancy, echocardiographic phenotyping of cardiac wasting in advanced cancer patients, the effect of sacubitril/valsartan for primary prevention of cancer therapy‐related cardiac dysfunction (CTRCD) in early breast cancer and the associations between centre volume and cardiogenic shock (CS) outcomes in Germany.

**Figure 1:**
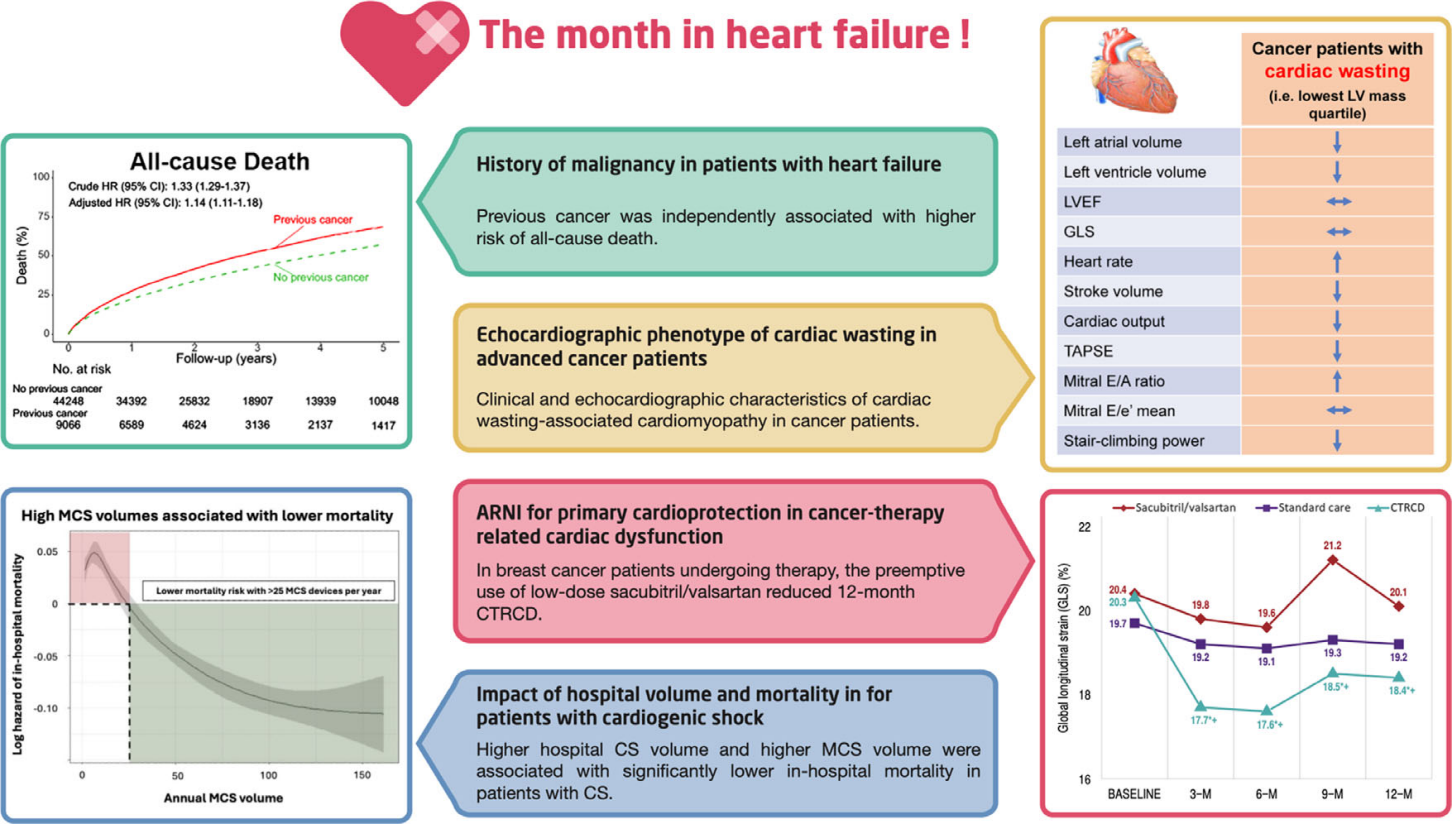
The month in heart failure – November 2025. CI, confidence interval; CS, cardiogenic shock; CTRCD, cancer therapy‐related cardiac dysfunction; GLS, global longitudinal strain; HR, hazard ratio; LV, left ventricular; LVEF, left ventricular ejection fraction; MCS, mechanical circulatory support; TAPSE, tricuspid annular plane systolic excursion.

## History of malignancy in patients with heart failure: insights from the Swedish Heart Failure Registry

Cancer and HF frequently coexist.^1^, ^2^ However, data on the clinical profile, treatment patterns, and outcomes of HF patients with a history of malignancy are scarce.^3^, ^4^

Ameri *et al*.^5^ analysed 53 314 patients from the Swedish Heart Failure Registry linked to the National Cancer Register between 2000 and 2020 to address this gap. Among these, 9066 patients (17%) had a prior cancer diagnosis made more than 2 years before HF diagnosis. The most prevalent malignancies were prostate (26%), breast (15%), colorectal (11%), and haematologic cancers (11%). Patients with a history of cancer were typically older, more often female, and exhibited a higher comorbidity burden, including atrial fibrillation, anaemia, and chronic obstructive pulmonary disease. Over a median follow‐up of 2.4 years, mortality rates per 100 patient‐years were higher in those with previous cancer (24; 95% confidence interval [CI] 23–25) compared with those without (18; 95% CI 18–19). Prior cancer was independently associated with an increased risk of all‐cause mortality (adjusted hazard ratio [HR] 1.14; 95% CI 1.11–1.18), non‐cardiovascular death (HR 1.38; 95% CI 1.31–1.44), and first all‐cause hospitalization (HR 1.11; 95% CI 1.09–1.14), whereas no association was observed with cardiovascular death or first HF hospitalization. Importantly, the excess risk of non‐cardiovascular death declined progressively with increasing time since the last cancer diagnosis. In patients with HF with reduced ejection fraction (HFrEF), a history of malignancy was associated with less frequent use of guideline‐directed medical therapy, including device therapy (cardiac resynchronization therapy/implantable cardioverter‐defibrillator).

In summary, this large, population‐based analysis highlights that a history of cancer is common in HF and associates with poorer non‐cardiovascular outcomes. Furthermore, HFrEF patients with prior malignancy appear to be undertreated with regard to guideline‐directed medical HF therapy. These findings emphasize the need for integrated cardio‐oncology care and careful consideration of including this high‐risk subgroup in future clinical trials.

## Echocardiographic phenotype of cardiac wasting in advanced cancer patients

Cardiac wasting‐associated cardiomyopathy is characterized by a loss of left ventricular mass and is an emerging concern in patients with advanced stage cancer.^6^ To this date a detailed assessment of the clinical features of cardiac wasting‐associated cardiomyopathy is missing. A detailed echocardiographic characterization may improve the understanding of the pathophysiology of this disease process and may further help to develop targeted therapies.^7^, ^8^

Anker *et al*.^9^ conducted a prospective study which included 398 patients with cancer hospitalized in the oncology wards of the Charité Campus Benjamin Franklin/Virchow Klinikum in Berlin between September 2017 and September 2023. All participants underwent a thorough clinical exam which included an echocardiographic examination with a follow‐up visit to investigate longitudinal changes in cardiac parameters. The authors found that patients with advanced cancer and low left ventricular mass exhibit a distinct echocardiographic phenotype which is characterized by lower cardiac chamber volumes, stroke volume, and cardiac output but normal left ventricular ejection fraction (LVEF) and global longitudinal strain (GLS). These characteristics may represent the distinct features of cardiac wasting‐associated cardiomyopathy.

The study confirms that patients with cardiac wasting‐associated cardiomyopathy suffer from symptoms that are similar to those of HF, as well as other adaptive changes such as lower stroke volume, higher heart rates, lower blood pressure, and more frequent anaemia. These findings may be of clinical relevance for cardio‐oncology trials, where cardiac wasting‐associated cardiomyopathy, specifically the loss of left ventricular mass over time, could be considered as a novel endpoint. Nonetheless, despite these findings it remains uncertain whether cardiac wasting‐associated cardiomyopathy is a distinct entity that contributes to poor outcomes or a further presentation of cachexia that involves both cardiac and skeletal muscle wasting.

## Sacubitril/valsartan for primary prevention of cancer therapy–related cardiac dysfunction in early breast cancer

Cancer therapy‐related cardiac dysfunction remains a common complication of curative breast cancer regimens. Although neurohormonal blockade has been explored, results have been inconsistent and definitions of CTRCD are heterogeneous. GLS is now recommended to detect subclinical cardiac injury.^10^ Hsu *et al*.^11^ conducted a phase II, randomized, open‐label, blinded‐endpoint trial testing low‐dose sacubitril/valsartan as primary cardioprotection during adjuvant therapy for early breast cancer.

Overall, 100 treatment‐naïve patients (mean age 50 years; 98% women) were randomized 1:4 to sacubitril/valsartan, initiated 3 days pre‐therapy and up‐titrated to 24.5/25.5 mg twice daily (*n* = 20), or standard care (*n* = 80) for 12 months. The primary endpoint (CTRCD defined as ≥15% relative GLS decline or ≥10‐percentage point LVEF drop to <50%) occurred in 0/19 patients in the sacubitril/valsartan arm versus 21/80 (26.3%) with standard care (*p* = 0.006; number needed to treat ≈3.8). Events were driven entirely by GLS declines, typically within 3–6 months. Secondary measures (LVEF, myocardial work indices) showed no between‐group differences overall, although the standard‐care CTRCD subgroup demonstrated transient LVEF decreases. Adverse events were infrequent. Two patients discontinued sacubitril/valsartan for hypotension.

These data suggest that pre‐emptive, low‐dose sacubitril/valsartan may attenuate subclinical systolic injury during modern breast cancer therapy. The study aligns with strain‐guided strategies (e.g. SUCCOUR) and responds to calls for proactive prevention in low‐to‐moderate risk cohorts.^12^ Nonetheless, the single‐centre design, small angiotensin receptor–neprilysin inhibitor cohort with unequal randomization, limited HER2 exposure, and 12‐month follow‐up limit generalizability. Further confirmation of the findings in adequately powered, multicentre phase III trials integrating GLS‐based endpoints, biomarker profiling, and patient‐centred outcomes is warranted.

## Impact of hospital volume and mortality in patients with cardiogenic shock and mechanical circulatory support

Cardiogenic shock is characterized by severe cardiac dysfunction resulting in systemic hypoperfusion and subsequent multiorgan failure. Despite significant advances in the treatment of myocardial infarction and HF, outcomes remain poor once these conditions progress to CS. Reported in‐hospital mortality rates range from 30% to 60%, depending on the underlying aetiology.^13^, ^14^, ^15^, ^16^

Dettling *et al*.^17^ examined the association between hospital case volume and in‐hospital mortality using data from 220 223 patients with CS treated across 1232 hospitals in Germany between 2017 and 2021. Hospitals were categorized according to their average annual CS and mechanical circulatory support (MCS) case volumes into high‐volume centres (upper tertile) and intermediate‐to‐low volume centres (lower two tertiles). Crude in‐hospital mortality rates were similar between intermediate‐to‐low volume (22 785/38658, 58.9%) and high‐volume centres (107 291/181 567, 59.1%). However, after adjustment for relevant confounders, treatment in high‐volume CS centres was associated with a significantly lower risk of in‐hospital death (HR 0.92, 95% CI 0.91–0.94; *p* < 0.001). Similarly, after adjustment for relevant confounders, treatment at high‐volume MCS centres was associated with a significantly lower in‐hospital mortality risk compared to intermediate‐to‐low volume MCS centres (HR 0.80, 95% CI 0.76–0.84; *p* < 0.001). Cubic spline analyses revealed a parabolic relationship between hospital CS volume and mortality, demonstrating a steep decline in mortality with increasing case volume up to approximately 90 CS cases per year, followed by a gradual rise in mortality beyond 300 cases per year, although mortality remained lower than at low‐volume centres. With regard to an MCS volume, greatest survival benefit was observed at centres performing >25 MCS cases annually. Alarmingly, 86% of hospitals utilizing MCS devices manage fewer than 25 cases per year.

These findings suggest that centralizing CS care within hub‐and‐spoke network structures, focusing on specialized high‐volume centres with established expertise in MCS management, may substantially improve patient outcomes.
